# Severe Hypophosphatemia After Zoledronate in a Young Adult With Malnutrition and Long-Term Parenteral Nutrition

**DOI:** 10.1016/j.aed.2025.08.006

**Published:** 2025-08-21

**Authors:** Tiffany Mach, Maria Wolfs, Mohammad Jay

**Affiliations:** 1 Division of Internal Medicine, Department of Medicine, McGill University, Quebec, Canada; 2 Division of Endocrinology and Metabolism, Department of Medicine, University of Toronto, Ontario, Canada

**Keywords:** zoledronic acid, bisphosphonate, hypophosphatemia, osteoporosis, total parenteral nutrition, malnutrition

## Abstract

**Background/Objective:**

Zoledronic acid, a third-generation bisphosphonate, is widely used to treat osteoporosis. Mild hypophosphatemia is relatively common after zoledronic acid, but severe cases are rare and under-recognized. We report a case of severe, sustained hypophosphatemia following zoledronic acid administration in a nutritionally vulnerable inpatient receiving long-term total parenteral nutrition. The objective of this report is to describe a patient who developed this complication, emphasizing nutritional vulnerability as an important risk factor.

**Case Report:**

A 31-year-old male with chronic malnutrition and prolonged total parenteral nutrition requirement due to complications of bowel disease was referred for assessment of multiple vertebral compression fractures. Baseline serum calcium, phosphate, magnesium, and renal function were normal. His 25-hydroxyvitamin D was 50 nmol/L and supplemented prior to zoledronic acid 4 mg intravenous administration. Two days later, his serum phosphate dropped to <0.30 mmol/L. Although asymptomatic, he required 5 days of intravenous phosphate replacement. Levels normalized by day 9.

**Discussion:**

This case illustrates that bisphosphonate-induced suppression of bone resorption, when superimposed on chronic phosphate depletion, recent nutritional repletion, and borderline vitamin D status may contribute to acute severe hypophosphatemia— even when baseline labs appear normal. In contrast to most previous reports involving malignancy or renal dysfunction, this case occurred in a patient with nutritional compromise and no history of malignancy or renal disease.

**Conclusion:**

Clinicians should recognize malnutrition, refeeding physiology, and total parenteral nutrition dependence as important risk factors for bisphosphonate-induced hypophosphatemia. Targeted phosphate monitoring and nutritional optimization should be considered before and after bisphosphonate therapy in nutritionally vulnerable individuals.

**Highlights**

• Severe low phosphate can occur after zoledronate in malnourished patients
• Normal baseline phosphate does not rule out risk after zoledronate use
• Long-term intravenous nutrition increases the risk of severe low phosphate levels
• Recent nutritional changes increase risk of zoledronate complications
• Consider monitoring phosphate before and after zoledronate in at-risk patients

**Clinical Relevance**

This case illustrates severe hypophosphatemia after zoledronate administration in a patient with malnutrition, requiring long-term TPN. It highlights the importance of phosphate monitoring before and after bisphosphonate therapy in high-risk patients, even when baseline laboratory results appear normal.

## Introduction

Zoledronic acid, a third-generation bisphosphonate, is widely used to treat osteoporosis due to its potent inhibition of osteoclast-mediated bone resorption.^1^ Mild and transient hypophosphatemia occurs in approximately 13% of patients following its intravenous administration, but severe hypophosphatemia is rare.^2^ Characterized as a serum phosphate level <0.30 mmol/L (<0.93 mg/dL), severe hypophosphatemia can lead to neurologic, cardiac, and muscular complications, including muscle weakness, paresthesia, seizures, arrhythmias, myocardial dysfunction, and altered consciousness.^3^^,^^4^

Most previously reported cases of bisphosphonate-associated hypophosphatemia have occurred in patients with malignancy, chronic renal disease, or primary hyperparathyroidism. Few have described this complication in patients without those underlying conditions, particularly in those with nonmalignant nutritional risk factors.^5^, ^6^, ^7^ We describe a case of severe hypophosphatemia following zoledronic acid administration in a hospitalized patient with chronic malnutrition, requiring long-term total parenteral nutrition (TPN).

## Case Report

A 31-year-old male was referred for evaluation of a nontraumatic T12 compression fracture with approximately 25% vertebral body height loss, which was incidentally found on plain radiographs. He had been hospitalized for over 2 years due to complications of stercoral colitis, including bowel perforation, fecal peritonitis, intra-abdominal abscesses, and entero-atmospheric fistulas. These required multiple bowel resections and led to significant gastrointestinal malabsorption, necessitating ongoing TPN for approximately 3 years. He had begun tolerating minimal oral intake about 1 month prior to bisphosphonate administration. His medications included calcium carbonate 1250 mg twice daily, vitamin D3 2000 IU daily, and pantoprazole 40 mg twice daily. He had no history of corticosteroid use, celiac disease, smoking, or alcohol intake.

## Diagnostic Assessment

Following the discovery of a T12 fracture, the patient underwent a vertebral fracture assessment and bone mineral density testing to evaluate underlying bone disease. The vertebral fracture assessment confirmed compression fractures at T12 and T5–T9. Bone mineral density revealed Z-scores of −4.6 (lumbar spine), −4.2 (left femoral neck), and −4.4 (left total hip), consistent with severely reduced bone density based on age.

Two main secondary causes of osteoporosis were considered: (1) chronic inflammation due to inflammatory bowel disease and recurrent infections, and (2) malabsorption secondary to multiple bowel resections. Mild hypogonadotropic hypogonadism was noted but untreated as it was presumed secondary to chronic illness. Other secondary causes of osteoporosis were excluded, including primary hyperparathyroidism, thyrotoxicosis, chronic renal disease, multiple myeloma, glucocorticoid exposure, and other confounding medications.

Given the presence of multiple fragility fractures and ongoing risk factors for bone loss, the patient was treated with intravenous zoledronic acid 4 mg. Baseline bloodwork prior to zoledronic acid administration showed normal ionized calcium, magnesium, phosphate, and renal function. His 25-hydroxyvitamin D level was 50 nmol/L, considered adequate by the Institute of Medicine’s threshold but suboptimal for bone health.^4^ With the goal of raising levels to ≥75 nmol/L, vitamin D supplementation was increased from 2000 IU to 3000 IU daily 1 week prior to treatment. However, no repeat vitamin D level was available at the time of infusion. Bone turnover marker C-terminal telopeptide was markedly elevated at 1605 ng/L (reference 225–936 ng/L), indicating high bone turnover. Parathyroid hormone (PTH) level was within normal range at 4.4 pmol/L (reference 1.3–9.3 pmol/L). Table 1 summarizes pretreatment laboratory data.

**Table 1:** Laboratory Investigations Prior to Initiation of Zoledronic Acid

Laboratory Investigations	Values	Reference Ranges
Ionized calcium	1.22 mmol/L	1.15–1.35 mmol/L
	(4.88 mg/dL)	(4.61–5.41 mg/dL)
Phosphorus	0.90 mmol/L	0.80–1.35 mmol/L
	(2.79 mg/dL)	(2.48–4.18 mg/dL)
Magnesium	0.86 mmol/L	0.63–0.94 mmol/L
	(2.09 mg/dL)	(1.53–2.28 mg/dL)
Creatinine	34 μmol/L	52–112 μmol/L
	(0.39 mg/dL)	(0.59–1.27 mg/dL)
Parathyroid hormone	4.4 pmol/L	1.3–9.3 pmol/L
	(41.36 pg/mL)	(12.26–97.70 pg/mL)
25-OH-Vitamin D	50 nmol/L	>50 nmol/L
	(20 ng/mL)	(>20 ng/mL)
Follicle-stimulating hormone (FSH)	6.8 mIU/mL	1.5–14 mIU/mL
	(6.8 IU/L)	(1.5–14 IU/L)
Luteinizing hormone (LH)	3.4 mIU/mL	1.4–7.7 mIU/mL
	(3.4 IU/L)	(1.4–7.7 IU/L)
Total testosterone	6.36 nmol/L	8.60–30 nmol/L
	(183.44 ng/dL)	(248.04–865.26 ng/dL)
Prolactin	30.1 μg/L	0–30 μg/L
	(30.1 ng/mL)	(0–30 ng/mL)
Thyroid-stimulating hormone (TSH)	1.52 mIU/mL	0.40–5.50 mIU/mL
	(1.52 IU/L)	(0.40–5.50 IU/L)
C-terminal telopeptide (CTX)	1127 ng/L	225–936 ng/L

Two days after zoledronic acid administration, bloodwork revealed severe hypophosphatemia (<0.30 mmol/L; reference 0.80–1.35 mmol/L), ionized hypocalcemia (1.04 mmol/L; reference 1.15–1.35), mild hypomagnesemia (1.06 mmol/L; reference 0.63–0.94 mmol/L), and normal creatinine (36 μmol/L; reference 52–112 μmol/L). PTH was elevated at 28.1 pmol/L (reference 1.3–9.3 pmol/L). The timeline of phosphate, calcium, and PTH levels after zoledronic acid administration is summarized in Figure. A 24-hour urine collection showed normal phosphate excretion and low calcium excretion. Repeat C-terminal telopeptide remained elevated (1127 ng/L), suggesting ongoing high bone turnover despite recent bisphosphonate therapy. The patient had no symptoms of hypophosphatemia, and electrocardiogram was normal. Post-treatment investigations are shown in Table 2.

**Fig:**
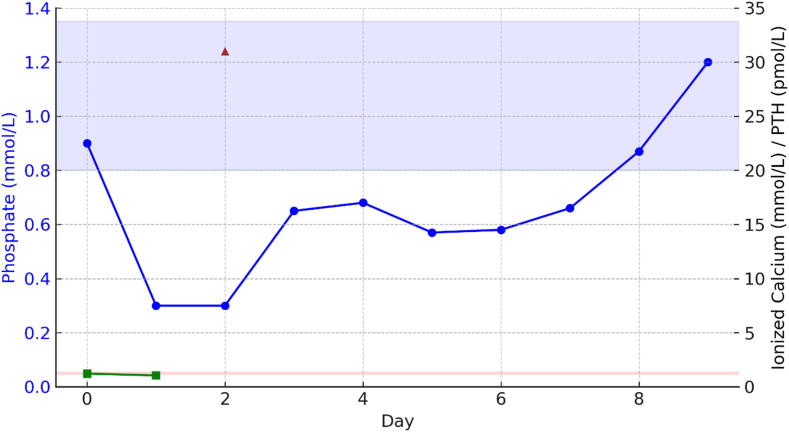
Trend of ionized calcium, phosphate, and parathyroid hormone (PTH) levels over time. Phosphate (blue line) is plotted on the left y -axis, with the shaded blue area indicating its reference range. Ionized calcium (green squares) is plotted on the right y-axis, with shaded pink areas indicating its reference ranges, and PTH (red triangles) is plotted without a reference range. Ionized calcium and PTH were only measured on select days, as shown. Day 0 represents the day of zoledronic acid administration.

**Table 2:** Investigations After the Initiation of Zoledronic Acid

Day	Phosphate (Reference 0.80–1.35 mmol/L) (2.48–4.18 mg/dL)	Ionized calcium (Reference 1.15–1.35 mmol/L) (4.61–5.41 mg/dL)	Magnesium (Reference 0.63–0.94 mmol/L) (1.53–2.28 mg/dL)	PTH (Reference 1.3–9.3 pmol/L) (12.26–97.70 pg/mL)	Creatinine (Reference 52–112 μmol/L) (0.59–1.27 mg/dL)
0^a^	0.90 mmol/L (2.79 mg/dL)	1.22 mmol/L (4.88 mg/dL)	0.86 mmol/L (2.09 mg/dL)	NR	34 μmol/L (0.39 mg/dL)
1	<0.30 mmol/L (<0.93 mg/dL) (nadir)	1.05 mmol/L	0.86–1.11 mmol/L	36 pmol/L	NR
2	<0.30 mmol/L (<0.93 mg/dL)	1.04 mmol/L (4.17 mg/dL)	1.06 mmol/L (2.58 mg/dL)	NR	36 μmol/L (0.41 mg/dL)
3	0.67 mmol/L (2.07 mg/dL)	NR	0.85 mmol/L (2.07 mg/dL)	NR	31 μmol/L (0.35 mg/dL)
4	0.71 mmol/L (2.20 mg/dL)	NR	1.06 mmol/L (2.58 mg/dL)	NR	32 μmol/L (0.36 mg/dL)
5	0.57 mmol/L (1.77 mg/dL)	NR	1.06 mmol/L (2.58 mg/dL)	NR	31 μmol/L (0.35 mg/dL)
6	0.59 mmol/L (1.83 mg/dL)	NR	0.84 mmol/L (2.04 mg/dL)	28.1 pmol/L (264.98 pg/mL)	32 μmol/L (0.36 mg/dL)
7	0.65 mmol/L (2.01 mg/dL)	NR	0.79 mmol/L (1.92 mg/dL)	NR	43 μmol/L (0.49 mg/dL)
8	0.89 mmol/L (2.76 mg/dL)	NR	0.88 mmol/L (2.14 mg/dL)	NR	45 μmol/L (0.51 mg/dL)
9	1.15 mmol/L (3.56 mg/dL)	NR	0.77 mmol/L (1.87 mg/dL)	NR	35 μmol/L (0.40 mg/dL)

Abbreviation used: PTH = parathyroid hormone.

a Day 0 refers to the date of zoledronic acid administration.

The patient had been receiving TPN since August 2021 due to chronic gastrointestinal losses and minimal oral intake. Prior to TPN initiation, serum phosphate was normal (0.74–1.22 mmol/L). While on TPN, phosphate levels fluctuated, with intermittent hypophosphatemia as low as 0.37 mmol/L. In April 2024, phosphate levels declined precipitously, reaching a nadir 2 days postzoledronic acid (<0.30 mmol/L).

## Treatment

Due to the limited oral intake, the patient was treated with intravenous sodium phosphate (15–30 mmol/d) and the sodium phosphate content of his TPN formulation was increased up to 30 mmol/d. At our institution, TPN is compounded by the pharmacy based on individualized daily orders prescribed by the clinical team. Electrolyte components, including phosphate, are adjusted based on daily serum values and clinical judgment. Proactive phosphate titration is not uniformly standardized across all patients, but in high-risk scenarios, TPN is often supplemented with higher phosphate content in anticipation of increased requirements. He required daily intravenous phosphate supplementation for 5 consecutive days after zoledronic acid administration. To mitigate secondary hyperparathyroidism, his calcium carbonate was increased to 2500 mg twice daily. Serum phosphate levels normalized 9 days after zoledronic acid initiation, and no further phosphate supplementation was required during his hospitalization.

## Discussion

This case describes a young adult with chronic malnutrition and malabsorption, requiring long-term TPN, who developed severe, sustained hypophosphatemia following intravenous zoledronic acid, despite normal baseline electrolytes. Hypophosphatemia is a known, but typically mild and transient, side effect of bisphosphonate therapy. It is most often reported in patients with malignancy, chronic renal disease, or hyperparathyroidism.^1^, ^2^, ^3^

Hypophosphatemia can result from transcellular shifts, reduced intestinal phosphate absorption, or increased renal phosphate excretion. The main regulators of serum phosphate concentration include: (1) PTH, which promotes phosphate excretion and bone deposition, (2) 1,25-dihydroxyvitamin D, which promotes intestinal and renal absorption, and (3) fibroblast growth factor-23, which enhances phosphate renal excretion.

Several mechanisms likely contributed to the severe hypophosphatemia observed in this patient.

1 Transcellular phosphate shifts: The patient had prolonged starvation and had only recently resumed partial oral intake, raising initial concern for refeeding syndrome. With refeeding, glycolysis stimulates intracellular phosphate uptake, leading to rapid depletion of extracellular stores. The timing of the hypophosphatemia, however, with the onset nearly a month following the initiation of oral intake, makes refeeding an unlikely primary contributor. It is more plausible that refeeding-related shifts led to earlier depletion of phosphate reserves, making the patient vulnerable to further decline triggered by zoledronic acid.
2 Decreased intestinal absorption: Limited oral intake, high ileostomy output, chronic malabsorption from bowel resections, chronic proton pump inhibitor use, and borderline vitamin D status likely contributed significantly to impaired phosphate absorption and low baseline reserves.
3 Renal phosphate handling: Conditions associated with increased renal phosphate wasting include hyperparathyroidism, vitamin D deficiency, diuretics, renal tubular acidosis, and rare genetic disorders. In this case, vitamin D was supplemented before treatment to enhance phosphate reabsorption, and 24-hour urinary phosphate excretion was normal. There was no history or biochemical evidence of hereditary disorders, Fanconi syndrome, or acute renal injury. Although PTH levels rose on day 4, baseline values were normal, suggesting a transient, compensatory response to hypocalcemia rather than a chronic elevation contributing to sustained phosphaturia.

Zoledronic acid’s effect on phosphate typically evolves over several days to weeks.^8^ In this case, chronic phosphate depletion from long-term TPN, refeeding physiology, and borderline vitamin D status created a vulnerable state with limited buffering capacity. In such a setting, even minimal phosphate shifts triggered by zoledronic acid were sufficient to precipitate severe hypophosphatemia.^9^

Unlike most reports of bisphosphonate-associated hypophosphatemia, which typically involve patients with malignancy, renal dysfunction, or hyperparathyroidism (Table 3), our case describes a young adult with chronic malnutrition without these typical risk factors. This case identifies a high-risk group that is often overlooked and highlights the importance of individualized risk assessment before bisphosphonate administration in patients with chronic malnutrition or recent nutritional repletion. A retrospective cohort study done by Ribeiro et al in 1992 demonstrated that patients receiving TPN in the intensive care unit had a ∼1.44-fold increased risk of developing hypophosphatemia compared to those on an oral diet (95% CI 1.10-1.89, *P* = 0.01) and parenteral nutrition alone was significantly more associated with hypophosphatemia than either enteral nutrition alone or parenteral plus enteral nutrition.^10^ Furthermore, a more recent prospective audit done by Pantoja et al in 2019 evaluated the incidence and management of refeeding syndrome in 80 patients initiated on TPN. Patients that were classified as high risk for refeeding syndrome received strict feeding protocols and despite adherence to this protocol, 30% of patients still experienced hypophosphatemia.^11^ These studies highlight the importance of monitoring electrolytes in high-risk patients on TPN who develop refeeding-related electrolyte disturbances, even with preventive strategies in place.

**Table 3:** Summary of Published Reports on Bisphosphonate-Associated Hypophosphatemia

Title	First author’s last name	Year	Bisphosphonate	Indication	Risk factors	Severity of hypophosphatemia
Multiple Electrolyte Abnormalities After Pamidronate Administration.	Elisaf	1998	Pamidronate 90 mg intravenously	Hypercalcemia of malignancy	Low baseline phosphorus levels Low magnesium	Moderate (values not available)
Medication-induced Hypophosphatemia: A Review.	Liamis	2010	Pamidronate	N/A	N/A	N/A
A Case of Severe, Prolonged, Refractory Hypophosphatemia after Zoledronic Acid Administration.	Clark	2016	Zoledronic acid 4 mg intravenously	Hypercalcemia of malignancy	Vitamin D deficiency Critical illness	Severe Phosphate levels dropped to 0.29 mmol/L (0.9 mg/dL).
Zoledronate Induced Hypocalcemia and Hypophosphatemia in Osteoporosis: A Cause of Concern.	Kaur	2016	Zoledronic acid	Osteoporosis	Full-text not available.	N/A
Profound and Protracted Hypophosphatemia after A Single Dose of Zoledronic Acid Infusion for Osteoporosis Associated with Normocalcemic Primary Hyperparathyroidism.	Chiam	2017	Zoledronic acid 5 mg intravenously	Post-menopausal osteoporosis and normocalcemic primary hyperparathyroidism	Primary hyperparathyroidism	Severe Phosphate levels dropped to 0.28 mmol/L (0.87 mg/dL).
Outcomes Following Intravenous Bisphosphonate Infusion in Pediatric Patients: a 7-year Retrospective Chart Review.	Nasomyont	2019	Zoledronic acid and pamidronate	Primary, secondary and glucocorticoid-induced osteoporosis	N/A	Mild to Moderate (No values available)
Hypocalcemia and Hypophosphatemia After Treatment with Zoledronic Acid in A Patient with AL Amyloidosis.	Tufano	2019	Zoledronic acid 4 mg intravenously	Metastatic bone disease	Chronic kidney disease Vitamin D deficiency	Moderate Phosphate levels dropped to 0.32 mmol/L (1 mg/dL).
Severe Hypophosphatemia Following Oral Bisphosphonate Treatment in a Patient with Osteoporosis.	Bagger	2020	Alendronate 70 mg orally weekly	Osteoporosis	Malabsorption syndrome	Severe Phosphate levels dropped to <0.16 mmol/L (0.5 mg/dL).
Drug-Induced Hypophosphatemia: Current Insights.	Megapanou	2020	Pamidronate and zoledronic acid	N/A	N/A	N/A
Life-threatening Hypophosphatemia Secondary to Zoledronic Acid Implementation in A Middle-Age Patient who Presented with Advanced Osteolysis in the Course of Multiple Myeloma.	Radziszewski	2020	Zoledronic acid (dose not specified)	Metastatic bone lesions.	Vitamin D deficiency Advanced osteolytic bone lesions	Severe Phosphate levels dropped to 0.2 mmol/L (0.62 mg/dL).

Severity was classified as mild, moderate, or severe based on reported nadir phosphate levels or clinical description when available.

One limitation is that urinary phosphate was measured after initiation of phosphate supplementation, possibly underestimating baseline renal phosphate losses. Additionally, fibroblast growth factor-23 and 1,25-dihydroxyvitamin D levels were not available, which limited detailed mechanistic insights into renal versus intestinal phosphate regulation, however, will be considered for future testing.

## Conclusion

This case expands the limited literature on bisphosphonate-induced electrolyte disturbances in nonmalignant, nutritionally compromised populations. Current guidelines do not recommend routine phosphate monitoring before bisphosphonate use. However, our case supports targeted screening in nutritionally vulnerable or hospitalized individuals. Given the increasing inpatient use of intravenous bisphosphonates, clinicians should adopt an individualized approach to phosphate monitoring and repletion in nutritionally vulnerable patients. In these groups, we propose that serum phosphate be assessed both before and after bisphosphonate administration.

## Statement of Patient Consent

Signed informed consent was obtained directly from the patient for the publication of this case report.

## Disclosure

The authors have no conflicts of interest to disclose.
